# Reverse breeding: a novel breeding approach based on engineered meiosis

**DOI:** 10.1111/j.1467-7652.2009.00450.x

**Published:** 2009-12

**Authors:** Rob Dirks, Kees van Dun, C Bastiaan de Snoo, Mark van den Berg, Cilia L C Lelivelt, William Voermans, Leo Woudenberg, Jack P C de Wit, Kees Reinink, Johan W Schut, Eveline van der Zeeuw, Aat Vogelaar, Gerald Freymark, Evert W Gutteling, Marina N Keppel, Paul van Drongelen, Matthieu Kieny, Philippe Ellul, Alisher Touraev, Hong Ma, Hans de Jong, Erik Wijnker

**Affiliations:** 1 Rijk Zwaan Breeding BVFijnaart, The Netherlands; 2 Max F. Perutz Laboratories, Department of Plant Molecular Biology, Vienna UniversityVienna, Austria; 3 Department of Biology, The Huck Institutes of the Life Sciences, The Pennsylvania State UniversityPA, USA; 4 School of Life Sciences, Institute of Plant Biology, Fudan UniversityShanghai, China; 5 Laboratory of Genetics, Wageningen UniversityWageningen, the Netherlands

**Keywords:** plant breeding, engineered meiosis, breeding per chromosome, asynapsis, univalent segregation, spore regeneration

## Abstract

Reverse breeding (RB) is a novel plant breeding technique designed to directly produce parental lines for any heterozygous plant, one of the most sought after goals in plant breeding. RB generates perfectly complementing homozygous parental lines through engineered meiosis. The method is based on reducing genetic recombination in the selected heterozygote by eliminating meiotic crossing over. Male or female spores obtained from such plants contain combinations of non-recombinant parental chromosomes which can be cultured *in vitro* to generate homozygous doubled haploid plants (DHs). From these DHs, complementary parents can be selected and used to reconstitute the heterozygote *in perpetuity*. Since the fixation of unknown heterozygous genotypes is impossible in traditional plant breeding, RB could fundamentally change future plant breeding. In this review, we discuss various other applications of RB, including breeding per chromosome.

## Introduction

One of the most important insights in plant breeding was the observation that hybrid (F1) progeny typically are superior in size, growth characteristics and yield in comparison to their homozygous parents, a phenomenon known as heterosis. Its underlying driving mechanisms may be multiple and are unfortunately poorly understood (Springer and Stupar, 2007; Stupar *et al.*, 2008; Fernandez-Silva *et al.*, 2009; Wei *et al.*, 2009). The unpredictable nature of heterosis confronts breeders with considerable difficulties: how does one optimize the performance of crop varieties when the constituents for success are unknown?

Breeders can evaluate heterosis by controlled crosses of inbred lines (i.e. by *apriori* selection and combination of unknown alleles). The hit-or-miss nature of this approach makes it difficult to optimize the effects of heterosis. Here, we propose an alternative strategy based on the reversal of crop selection: the generation of defined populations with high levels of heterozygosity and random variation. These populations are then assessed in a variety of environmental conditions (latitude, salinity, humidity, etc.) and the best performing heterozygous germplasm is selected for further breeding.

A barrier to achieving high levels of variation in current plant breeding programs is that uncharacterised heterozygotes are difficult—if not impossible—to reproduce by seeds. Favourable allele combinations of the elite heterozygote are lost in the next generation due to segregation of traits. Because of this difficulty, the development of methods for easy preservation of heterozygous genotypes is one of the greatest challenges in plant breeding. Apomixis has repeatedly been proposed as a way to preserve heterozygous phenotypes, but has not yet led to breeding applications (Perotti *et al.*, 2004).

In this paper, we show how a new technique, reverse breeding, meets the challenge of fixation of complex heterozygous genomes by constructing complementing homozygous lines (Dirks *et al.*, 2003). This is accomplished by the knockdown of meiotic crossovers and the subsequent fixation of non-recombinant chromosomes in homozygous doubled haploid lines (DHs). The approach not only allows fixation of uncharacterized germplasm but provides breeders with a breeding tool that, when applied to plants of known backgrounds, allows the rapid generation of chromosome substitutions that will facilitate breeding on an individual chromosome level. After a brief introduction to the RB breeding scheme, we first elaborate on the basis of RB: the unique character of achiasmatic meiosis. Thereafter, we show how the technique may be implemented in crops followed by a discussion of its main applications.

## Reverse breeding

**Figure 1:**
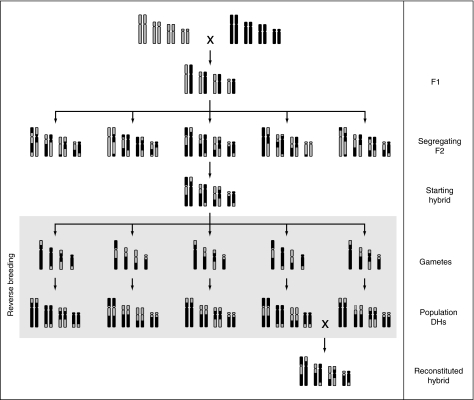
Reverse breeding can be used to fix unknown heterozygotes. Crossing two homozygous parents (grey and black bars) creates a heterozygous F1. When selfed, the F1 produces a segregating F2 population. A starting hybrid of unknown genetic constitution is selected for its desireable characteristics, and subjected to the two steps of reverse breeding (grey box). By knocking down meiotic crossing over, whole parental chromosomes are transmitted through spores, without rearrangement. Note, in this example the four chromosomes in the hybrid can generate 16 different combinations in the gametes—only five are shown for convenience. The achiasmatic gametes are then used produce doubled haploid (DH) lines using *in vitro* culture techniques. From this population, complementary parents can be chosen that when crossed perfectly reconstitute the starting hybrid. The DH lines then serve as a permanent library that can be used to predictably generate a wide variety of defined hybrids.

Reverse breeding comprises two essential steps: the suppression of crossover recombination in a selected plant followed by the regeneration of DHs from spores containing non-recombinant chromosomes. Figure 1 shows an idealized crossing scheme that employs RB. It depicts the generation of a segregating population (in this case a segregating F2), from which a genotypically uncharacterized plant with a favourable combination of traits is selected. Crossing over is suppressed in this plant and achiasmatic gametes are collected, cultured, and used to generate DHs. The DH lines can then be used to recapitulate the elite heterozygote on a commercial scale.

**Figure 2:**
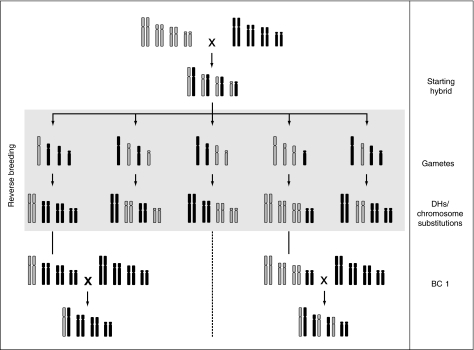
Reverse breeding can be used as advanced breeding tool. As starting hybrid for a reverse breeding experiment, a fully heterozygous F1 is chosen, resulting from a cross between two homozygous parents. Application of reverse breeding (grey box) leads to a population of doubled haploids. Note that among those DHs, there are chromosome substitution lines of one of the starting parents into the backgroud of the other. Lower left: a chromosome substitution line for a grey chromosome in the black parent can be backcrossed with the fully black parent to create a hybrid that is heterozygous for just one chromosome. Such hybrids serve as starting point for breeding per chromosome (explained in text). Lower right: a chromosome substitution line for a black chromosome in the grey parent can be backcrossed with the fully black parent to create a hybrid that is homozygous for just one chromosome. Such hybrids are starting points for studying background interactions (explained in text).

In another application, RB can be applied to plants of known background (Figure 2). If crossing over is eliminated in the F1 hybrid rather than the F2 generation, RB can be used to generate chromosome substitution lines. These lines contain one or more chromosomes from one parent in the background of the other parent. By backcrossing the chromosome substitution lines to the original parental lines, one can obtain populations that segregate only for the heterozygous chromosome(s). Reverse breeding, in theory, allows the re-shuffling of chromosomes between two homozygous plants in all possible ways.

## Reverse breeding relies on achiasmatic meiosis

### On the function of crossovers

**Figure 3:**
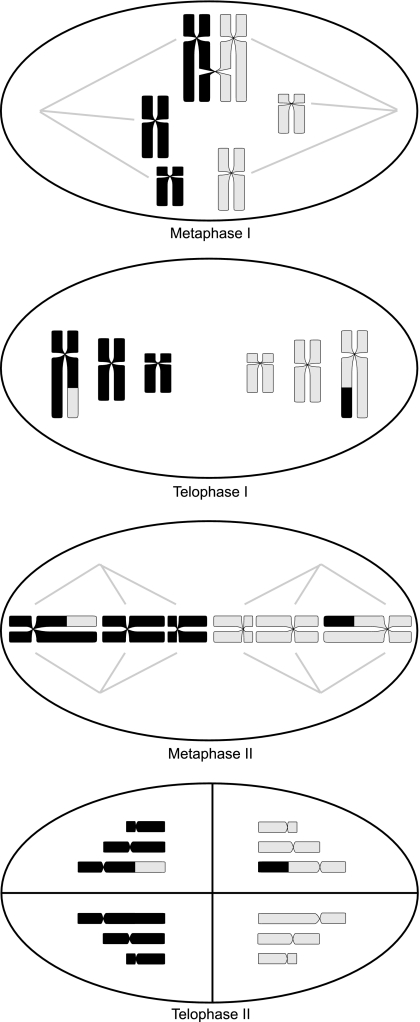
The presence of a single crossover in a chromosome pair does not affect the utility of reverse breeding. The figure depicts four cells at different stages of meiosis. At metaphase I, a single crossover is present in one chromosome pair (a bivalent pair) whereas other homologues remain as univalents. The homologues joined by a chiasma segregate to opposite poles and—in this example—the univalents segregate randomly to opposite poles, giving rise, in this case, to a balanced dyad (at telophase I). Meiosis then proceeds through metaphase II, separating sister chromatids, and at telophase II four gametes are formed. Half of these gametes contain a recombinant chromosome (upper two), whereas the other half contain non-recombinant chromosomes (lower two) and are useful for reverse breeding.

In flowering plants, the formation of crossovers during meiotic prophase I relies on synapsis, the extensive and stable interaction between homologous chromosomes, mediated by a complex proteinacious structure called the synaptonemal complex (Moses, 1956). During crossing over, two homologues become physically joined when the distal end of one chromatid is attached to the proximal end of a non-sister chromatid and vice versa (Figure 3). The resulting intermediate of joined homologues is called a bivalent. Crossover sites are visible as cross-like structures after synaptonemal complex disassembly, the chiasmata. They are usually maintained until metaphase I/anaphase I, when homologues segregate to opposite poles. In most plants, a chromosome pair typically has one or two crossovers. Many mutants have been described that reduce or eliminate crossovers (reviews in Hamant *et al.*, 2006; Noyes *et al.*, 2006; Roeder, 1990; Zickler and Kleckner, 1999).

Achiasmatic chromosomes (chromosomes that did not form crossovers) remain as univalents (Figure 3). Chiasmata, that in bivalents promote segregation of homologues to opposite poles (regular disjunction), are absent in univalents and the homologues may segregate to the same pole instead (non-disjunction). This leads to unbalanced chromosome numbers (aneuploidy) in the spores. Consequently, achiasmatic plants are highly sterile (Couteau *et al.*, 1999; Hartung *et al.*, 2007). The more univalents are present, the more aneuploid pollen are formed. Assuming that each univalent has an equal chance of moving to either pole, the probability of a spore with a normal chromosome complement is ½^*x*^, with *x* equalling the haploid chromosome number of the species. Hence, for *Arabidopsis* (2*n* = 2*x* = 10), the frequency of balanced spores is one in 32 (3%). For plants with a basic chromosome number exceeding 12, the chances of finding balanced gametes get very (perhaps too) small (with only one out of over 4000 spores being balanced). This has to be evaluated for individual crops. In the case of *Petunia* with an estimated number of 30 000 microspores per anther (Kapoor *et al.*, 2002) and seven chromosome pairs, we expect that the number of euploid spores per anther will be 235. *Arabidopsis* by comparison only produces 2800 spores per flower on average (Noyes *et al.*, 2006) and will generate 88 euploid spores assuming no bivalent formation and random chromosome distribution.

### Chances of finding complementing parents

The maximum number of different DHs obtained from a heterozygous diploid in a RB experiment equals 2^*x*^, with *x* being the basic chromosome number. The probability that two DHs form a pair of ‘complementary’ parents (as shown in Figure 2) equals 2^*x*^/(2^*x*^)^2^ = (½)^*x*^, and the probability that they, upon crossing, do not reconstruct the original genotype is 1−(½)^*x*^ = (2^*x*^−1)/2^*x*^. The number of combinations between different DHs, presuming that reciprocal crosses result in the same phenotype, is *n*(*n*−1)/2. In the case of *n* DHs, the probability of not finding a complementary pair of lines is therefore [(2^*x*^−1)/2^*x*^]^*n*(*n*−1)/2^ and the probability of at least one complementary combination of two DHs is given by the formula [(2^*x*^−1)/2^*x*^]^*n*(*n*−1)/2^ = 0.01 (*P* = 99%). This equation can be solved for different values of *x*. The number (*n*) of DHs that must be generated for finding a complementary match is highly dependent on the haploid chromosome number (*x*) and is given in Table 1.

**Table 1:** Number of non-recombinant DHs required for reconstructing the original starting plant at different probability levels in various species

	Probability				
Haploid chromosome number (*x*)	0.90	0.95	0.99	1.00	Model species/crop
5	13	14	18	47	*Arabidopsis*
6	18	20	25	67	Spinach, corn salad
7	25	28	35	94	Cucumber
8	35	40	49	133	Onion
9	49	56	69	188	Barley, carrot, sugarbeet, most vegetable Brassicas, lettuce
10	69	79	98	266	Maize, sorgum, asparagus, cocoa
11	98	111	138	377	Banana, watermelon, celery, fennel, common bean
12	138	157	195	532	Tomato, pepper, melon, rice, egg plant

## The technical realization of reverse breeding

### Effective suppression of recombination

Reverse breeding relies on the effective suppression of meiotic crossovers. Therefore, genes that are essential in crossover formation but leave the chromosome structure intact are particularly useful. Examples are the *Arabidopsis ASY1* and the rice *ASY1* homologue *PAIR*2, the mutants of which display univalents at metaphase I (Ross *et al.*, 1997; Caryl *et al.*, 2000; Nonomura *et al.*, 2004). Other mutants with similar phenotypes are *dmc1*, *sds*, *ptd* and *spo11* (Couteau *et al.*, 1999; Azumi *et al.*, 2002; Stacey *et al.*, 2006; Wijeratne *et al.*, 2006).

The knockdown of gene expression, essential for RB, can be achieved by targeting genes using RNA interference (RNAi) (as shown by Siaud *et al.*, 2004; Higgins *et al.*, 2004) or siRNAs, which will result in predominantly post-transcriptional gene silencing (PTGS). Alternatively, dominant-negative mutations of the target gene can be used. The human meiotic recombination protein DMC1 forms octomeric rings (Kinebuchi *et al.*, 2004), but is fully defective in both ssDNA and dsDNA binding activities, when an amino terminal deletion lacking 81 amino acids is made (Kinebuchi *et al.*, 2005). Similar dominant-negative alleles of *DMC1* resulted in loss of male meiotic recombination in mice (Bannister *et al.*, 2007).

In crops in which stable transformation is difficult or impossible to achieve, other techniques could be applied. Virus-induced gene silencing (VIGS) was shown to be an effective technique for induction of PTGS. A plant then is infected with a virus that was modified to include a target gene RNA sequence. In a defence reaction, the plant will break down the viral RNA using siRNA, targeting simultaneously the plants’ endogenous mRNA (Ruiz *et al.*, 1998; Baulcombe, 2004). Alternatively, target genes may be silenced by silencing molecules delivered by graft transmission (Shaharuddin *et al.*, 2006). Shoots of the plant in which genes are to be silenced would be grafted on transgenic rootstocks. In this case, only few transgenic rootstocks would be required to routinely apply RB in many crops. Another more recent approach is based on a forward chemical genetic screen that identified ‘mirin’ as an inhibitor for the Mre11-Rad50-Nbs1 complex (Dupré*et al.*, 2008). Exogenous application of compounds that cause inhibition or omission of recombination during meiosis would speed up the application of RB enormously.

A major advantage for using chemicals that repress crossovers or graft transmission of silencing molecules is that the resultant RB products (DHs) are free of transgenes. This is important, because the RB products are destined to be used in further breeding schemes, and should not have a achiasmatic phenotype. Perhaps contrary to intuition, DHs produced by transgene-mediated methods can be transgene free. If a dominant knock-down construct is present in hemizygous state, half of the spores that are formed will not carry the transgene and, hence, are non-transgenic. Multiple transgenic lines with knock-down constructs on different chromosomes can be used to generate a full array of complementary DHs that do not carry transgenes (Wijnker and de Jong, 2008).

Crossover suppression need not be complete to be useful for RB. It can be explained that a single residual crossover may still occur in any chromosome pair(s). A single crossover causes regular segregation of the homologues involved (thereby increasing the chance of obtaining a balanced gamete twofold). A crossover also generates two recombinant chromatids, which are not useful for RB. But since a crossover affects only half of the chromatids of the bivalent pair, the other two chromatids are non-recombinant, and useful. Consequently, half of the resulting spores are potentially useful for RB (Figure 3). In short, residual crossovers (provided there is only one per bivalent) increase the incidence of DHs carrying recombinant chromosomes, but still produce 50% of spores carrying non-recombinant chromosomes. These non-recombinant spores can be selected for by using molecular markers.

### Doubled haploids

Doubled haploid plants resulting from achiasmatic meiosis can be obtained from unfertilized ovules (gynogenesis) or from microspore and anther cultures (androgenesis), according to well-established protocols that have been developed for a variety of plant species including crops (Jain *et al.*, 1996). The efficiency of DH formation from haploid spores is species dependent (Forster *et al.*, 2007). The unique characteristic of DHs made from spores produced through achiasmatic meiosis is explained in Figure 1: they contain non-recombinant parental chromosomes. Note however that aneuploid unfertile spores, which are in fact most prevalent, were not depicted. Selection of the required euploid spores is in part automatically achieved since only spores containing at least one copy of all chromosomes can pass through all developmental stages, from cell division and embryogenesis to plant regeneration. Hyperploid offspring could be selected against using co-dominant markers or flow cytometry.

Development of RB is limited to those crops where DH technology is common practice. For the great majority of crop species this technology is well established and professional breeding companies routinely use such techniques in their breeding programs (Maluszynski *et al.*, 2003; Forster *et al.*, 2007). There are, however, some notorious exceptions such as soybean, cotton, lettuce and tomato where doubled haploid plants are rarely formed or not available at all (Croser *et al.*, 2006; Segui-Simarro and Nuez, 2007; Zhang *et al.*, 2008). Genotyping of DHs by molecular markers is routine practice in contemporary plant breeding (De Vienne, 2003) and is also indispensable for RB. In the complete absence of meiotic recombination one polymorphic molecular marker per chromosome would suffice to genotype every DH since the entire chromosome would behave as a single linkage block. In the presence of any residual crossovers, two markers (as distally located as possible) are required per chromosome.

## Reverse breeding applications

### Reconstruction of heterozygous germplasm

For crops where an extensive collection of breeding lines is still lacking, RB can accelerate the development of varieties. In these crops, superior heterozygous plants can be propagated without prior knowledge of their genetic constitution (also see Figure 1). Table 1 shows the number of doubled haploid plants that are necessary to reconstruct the starting plant at different levels of probability. The number of DHs that is required is surprisingly low. For instance in maize (*x* = 10) just 98 DHs are expected to contain a set of two reciprocal genotypes (*P* = 99%).

### Breeding on the single chromosome level

Many interesting characteristics in crops are based on polygenic gene interactions, very often located on different chromosomes. These quantitative traits are therefore not easy to breed on. Figure 2 explains how chromosome substitution lines can be obtained when RB is applied to an F1 hybrid of known parents. These homozygous chromosome substitution lines provide novel tools for the study of gene interactions. When crossed with one of the original parents, hybrids can be formed in which one of the chromosomes is homozygous (Figure 2, lower right), whereas it is also possible to produce hybrids in which just one chromosome is heterozygous (Figure 2, lower left). The former allows the study of epistatic interactions between the background and genes contributed by the substitution chromosome. Offspring of plants in which just one chromosome is heterozygous, will segregate for traits present on that chromosome only. Selfing plants that carry a substituted chromosome (or using recurrent backcrosses) will allow breeders to fine-tune interesting characteristics on a single chromosome scale. This could bring forth improved breeding lines carrying introgressed traits. The few examples were shown here demonstrate that RB presents breeders with full control over homo- or heterozygosity at the single chromosome level.

Note that finding specific substitution lines may be difficult, since they are rare occurrences. Depending on the efficiency of the DH system, especially crops with high chromosome numbers may pose problems. In these cases backcrossing a DH line carrying the desired substitution in addition to another (undesired substitution) with one of the original parents may be helpful. Using marker assisted breeding the desired chromosome substitution can be obtained with relative ease.

### Reverse breeding and marker assisted breeding

Especially in combination with (high throughput-) genotyping, reverse breeding becomes a versatile tool. Evidently, high throughput genotyping speeds up the process of identification of complementing parents in populations of DHs in early stages. But perhaps more powerful is its use in the study of gene interactions of the various heterozygous inbred families (HIFs) that can be produced by crossing and backcrossing the products of RB (as was explained above). The screening of populations that segregate for traits on a single chromosome allow the quick identification of QTLs, when genotyping is combined with –for example- transcriptome or metabolome profiling. Such HIFs further aid the generation of chromosome specific linkage maps and the fine mapping of genes and alleles. RB can as such provide highly valuable insights into the nature of heterotic effects.

### Backcrossing in CMS back ground

In several vegetable crops such as cabbages and carrots, breeders make use of cytoplasmic male sterility (CMS) (Chase, 2007). In these systems, the presence of male sterility presents a special challenge to RB. In these cases, gynogenesis rather than androgenesis can be used to obtain DH plants. This is perfectly compatible with RB in the sense that the chromosomes from the maintainer line can be recovered directly in the cytoplasm of the sterile line in one step. Gynogenesis has been described in several crops such as *Brassica*, maize, sugar beet, cucumber, melon, rice, onion, sunflower, and barley (Keller and Korzun, 1996). However, the development or improvement of the protocol for many species was often abandoned when anther and microspore culture techniques were developed. In cases where the efficiency of gynogenesis is too low, it is possible to cross the male sterile (A) lines with maintainer lines (B) that carry one copy of a restorer gene. The AB combination will be fertile and RB can be performed. In rice, restorer genes have been successfully transformed (Wang *et al.*, 2006). It should therefore be possible to use a restorer gene and a gene for crossover suppression in the same vector (both transgenes) and perform RB in a ‘double suppressed’ (CMS and crossover) background.

## Conclusions

The combination of crossover suppression, followed by the regeneration of haploid spores into DHs results in novel and powerful breeding applications. One important application is the production of complementary homozygous lines that can be used to generate specific F1 hybrids. Additionally, when RB is applied to F1 heterozygotes, it is possible to generate chromosome substitution lines that allow targeted breeding on the single chromosome scale. RB is fully compatible with commercial CMS lines that are frequently used in modern agriculture.

The technique however is limited to crops with a haploid chromosome number of 12 or less and in which spores can be regenerated into DHs. In polyploids or species with high chromosome numbers, another reconstruction method has been proposed that is based on the omission of the second meiotic division, leading to unreduced second division restitution (SDR) spores. The use of these SDR spores enables the near reconstruction of desired phenotypes, and also provides the possibility of obtaining chromosome substitution lines (Van Dun and Dirks, 2006).

There is growing interest in the development of plant breeding techniques that are based in modifications of meiosis (Wijnker and de Jong, 2008). However, most techniques are merely extensions of the ‘classic’ plant breeding practice aimed at more efficient introgression of traits from alien backgrounds into crops. Pivotal for understanding the expected impact of germplasm fixation on plant breeding should be the realization that plant breeding relies heavily on the human eye for the selection of breeding lines. It is not difficult to imagine that selection for (overdominant) complex traits or QTLs is a daunting task. Visual selection is therefore always accompanied by extensive testcrosses aimed at control avoiding the loss of valuable traits during selection. Methods that allow the fixation of elite germplasm (apomixis and reverse breeding) provide alternatives to this selection process. Though reverse breeding may appear more complex than apomixis at a first glance, it does not suffer from the drawback of the current knowledge of apomixis where the three mechanisms essential for apomixis (apomeiosis, parthenogenesis and endosperm formation) have to be operational and synchronized (Koltunow and Grossniklaus, 2003). As a plant breeding tool, reverse breeding may be regarded more versatile as its controlled deconstruction of complex genotypes into homozygous parental lines allows the further improvement of these lines by classic breeding methods.
